# An Experimental Investigation of the Mechanical Behavior and Particle Crushing Characteristic of Volcanic Soil

**DOI:** 10.3390/ma15155423

**Published:** 2022-08-06

**Authors:** Xiao-Yang Liu, Chang-Ming Wang, Hai-Liang Liu, Di Wu

**Affiliations:** College of Construction Engineering, Jilin University, Changchun 130026, China

**Keywords:** volcanic soil, mechanical property, strength index, particle crushing, road engineering

## Abstract

Volcanic soil is a special soil that is well-known for its distinctive texture, vesicular nature, and particle fragility. The fragility characteristic of volcanic soil is the main factor affecting the foundation stability in road engineering. This study focuses on the mechanical properties and particle crushing characteristics of volcanic soil retrieved from Northeast China. A series of triaxial consolidation and drainage shear tests are performed on volcanic coarse-grained soil (5 mm > d > 0.075 mm) under different initial relative densities and effective confining pressures. Results show the peak friction angle of volcanic soil significantly decreases with the increase of confining pressure. The particle crushing degree of volcanic soil increases with the increase of confining pressure, particle size, and relative density. The relative breakage rate of the same particle size group has a good linear relationship with a fractal dimension. Moreover, for the same particle size, the relationship between plastic work and relative breakage rate can be fitted by a power function, which is not significantly affected by relative density or effective confining pressure. From an engineering view, in addition to increasing the compaction degree of volcanic soil, volcanic soil with fine particles used as a roadbed filler can significantly reduce the deformation of the roadbed and improve the bearing capacity of the foundation.

## 1. Introduction

In recent years, with the increasing expansion of road construction, some roads have to cross volcanic soil areas, considering local availability in large quantities and low cost [1], the use of volcanic soils in roadbed fill has increased.

As a regional special soil, volcanic soil differs from traditional sand and gravel stacks in that its genesis originates from the eruption and condensation of volcanic magma. This special genesis allows volcanic soils to play different roles in various fields, such as refractory materials and adsorbents to remove harmful ions from industrial wastewater [2,3,4,5,6,7]. Mechanical properties are the focus of the present study. With the ejection of magma, under the conditions of reduced external pressure and a sudden temperature drop, gas escapes from the lava and forms pores of various sizes and numbers, moreover, since this material has not undergone long-distance transport effects, as a result, volcanic soil exhibits porous characteristics and irregular particle shape characteristics [8]. These special structures make the volcanic soil particle easy to crush under lower loads [9,10], this causes excessive deformation even rapid weakening of volcanic soil foundation bearing capacity, leading to instability and other hazards, affecting the safe operation of road facilities. Therefore, an in-depth investigation of volcanic soil particle crushing characteristics is meaningful for the stability of road engineering.

The special crushing sensitivity of volcanic soils has attracted the attention of scholars. According to the results of previous studies, particle crushing is related to a variety of factors, mainly including mineral composition [11,12,13], particle shape [14,15,16], particle size [17,18,19], particle relative density [20,21], and external loads [22,23], etc. Extensive research has been performed to study the mechanical properties of crushable volcanic soils retrieved from different regions. For example, Agustian and Goto [9] investigated the effects of particle size, dry density, and effective confining stress on Japanese volcanic soils by drained triaxial compression tests and found particle sizes had a noticeable influence on internal friction angle. Kikkawa et al. [24] performed K0 compression tests to compare the compression behavior of loose and dense sand and pointed out that dense sand particles exhibited more crushing and less tendency during loading. Galvis-Castro et al. [25] conducted a one-dimensional compression study of volcanic soils in the Columbia area, the result showed that yield stress was low. Asadi et al. [26] reported a crushable volcanic soil acquired from the North Island of New Zealand and found its shear modulus was much lower than Toyoura sand. Scholars’ studies show that the mechanical strength of volcanic soil was low due to its fragile crushing nature. Although scholars have achieved plenty of achievements in crushable volcanic soil. However, quantitative studies on particle crushing characteristics and the mechanical behavior of volcanic soils are not sufficient, such as quantitative measurement of particle crushing, the relationship between the crushing of volcanic soil particles and the external input energy, etc. A deeper study of these aspects will help to better understand the mechanical properties of crushable volcanic soils and provide theoretical guidance for the construction in road engineering.

Given this, the objective of this study is to investigate particle size (*d*), confining pressure (*σ*_3_), and relative density (*Dr*) on the mechanical behavior and crushing characteristics of volcanic soils. The mechanical characteristics, strength indexes, and critical state of volcanic soils were examined. Then, particle crushing features after the shearing test was addressed using the quantitative indexes relative breakage rate and fractal dimension, and the particle crushing pattern was analyzed. Finally, from an energy perspective, the relationship between plastic work and relative breakage rate was established.

## 2. Materials and Methods

### 2.1. Material

**Figure 1:**
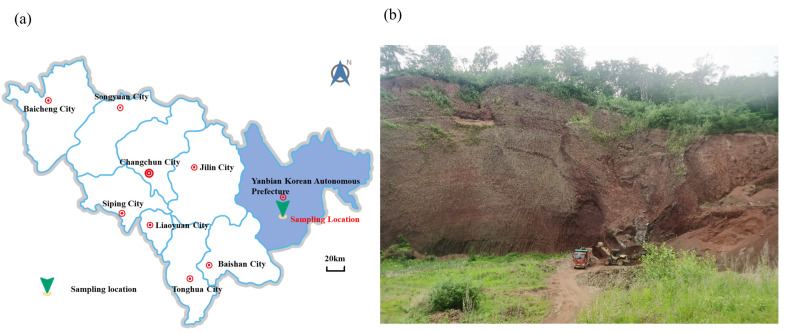
Sampling location map. (**a**) Location map; (**b**) Artificial slope.

**Figure 2:**
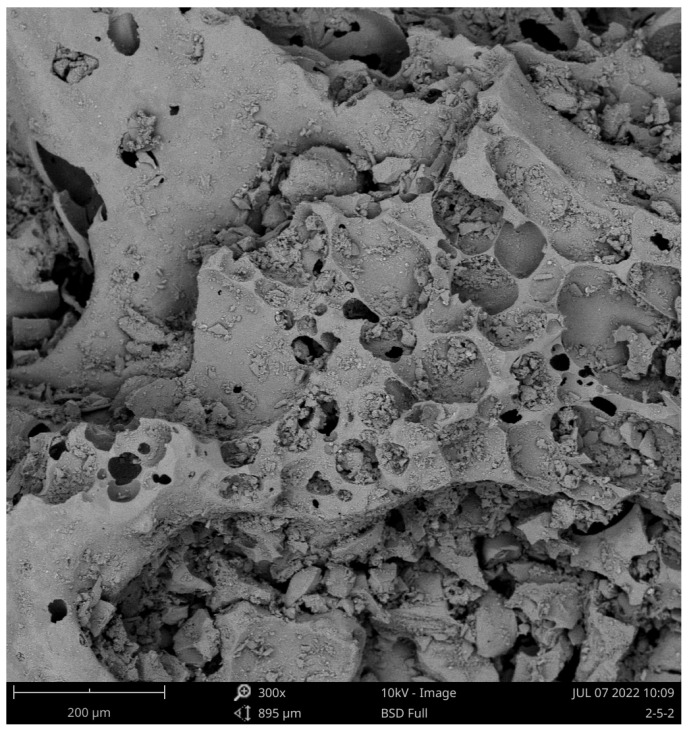
SEM of volcanic soil particle.

The soil sample material was taken from Erdao Baihe District, Yanbian Korean Autonomous Prefecture, Jilin Province, China (Figure 1a). Thick layers of volcanic soil were formed at this site due to the eruptive action of Changbai Mountain; the sampling site was located at a slope formed by artificial excavation (Figure 1b). There are numerous small pores distributed inside the volcanic soil particles (Figure 2), hence this material is susceptible to crushing under external forces, distinguishing it from ordinary materials [27].

The mineralogical and chemical composition of volcanic soil were analyzed by X-ray diffraction (XRD) and X-ray fluorescence (XRF). (JASSO TTR III Multifunctional X-ray Diffractometer was adopted for the XRD test. The K-value method was used to calculate mineral content. M4 Tornado X-Ray Fluorescence Spectrometer was used for the XRF test. For more test details, please see the literature [28]). Table 1 shows the mineralogical composition of volcanic soil consisting mainly of quartz, feldspar (potassium feldspar and plagioclase), and hematite with little clay minerals. The chemical composition is dominated by SiO_2_.

### 2.2. Test Scheme

**Figure 3:**
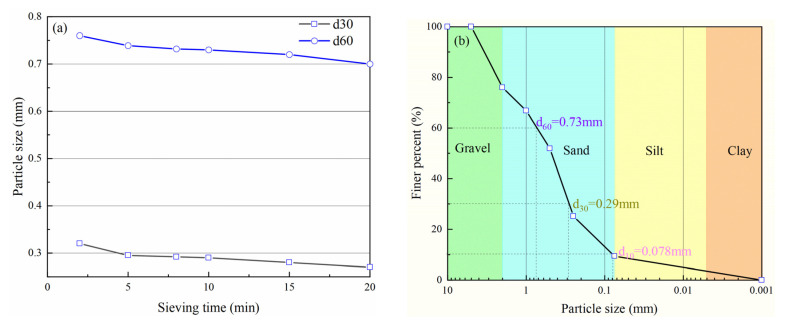
Determination sieving test time and corresponding particle size distribution curve: (**a**) sieving test time; (**b**) particle size distribution curve.

**Figure 4:**
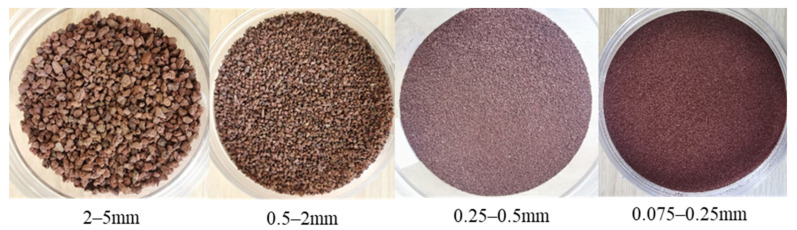
Diagram of four particle size of volcanic soil.

Due to the irregular shape and fragility of volcanic soil particles, the soil particle crushing degree increases with the increase of sieving time. This will affect the accuracy of the result of particle size distribution curves. To overcome the effect, a suitable sieving time needs to be determined. Figure 3a shows that with an increase in sieving time, control particle size *d*_30_, *d*_60_ gradually decrease, when the sieving time is within 8–10 min, control particle size change is not significant so sieving time is determined as 10 min. Figure 3b is the original curve in sieving 10 min. According to Hardin’s theory [11], it is difficult for a soil particle size < 0.074 mm to crush. Considering particle size groups engineering classification, four particle size groups were selected from original grading curves, which all belong to the category of coarse-grained soils [29]. The schematic diagram of the four particle sizes group is shown in Figure 4. The physical properties of volcanic soil were determined according to the Standard for Geotechnical Testing Method (2019) [30]. Combined with Figure 4 and Table 2, it can be found that for the 2–5 mm particle size group and 0.5–2 mm particle size group, particle shape is irregular and the maximum void ratio is relatively large with holes visible to the naked eye.

As volcanic soil has favorable permeability properties due to its porous nature [31], water can be discharged quickly under load, therefore consolidation and drainage test conditions are realistic. Considering that general road engineering load values are not large, effective confining pressures were set as 100 kPa, 200 kPa, and 400 kPa, and three defined in engineering relative densities (loose (0.3), medium-dense (0.5), and dense (0.7)) were selected in this paper to carry out consolidation drainage shear (CD) experiments, the test protocol is shown in Table 3.

### 2.3. Triaxial Experiment Procedure

The tested soil samples were 50 mm in diameter and 100 mm in height. A 0.5 mm thick rubber film was chosen to avoid particles from penetrating the rubber film. The soil was divided into five parts, and it was carefully dropped into rubber film embraced using a spoon. To avoid massive particle crushing during the sample loading stage, a sample maker was used to gently compress the sample surface to the preset height. Then, the sample was saturated by combining the water head and the backpressure saturation method. In the water head saturation stage, the sample was saturated for 2.0 h, and a back pressure of 400 kPa was applied to the sample in the backpressure saturation stage; when B-value > 0.95, the saturation stage finished and the sample was consolidated under the specified effective confining pressure; when the change of consolidation volume did not exceed 0.05 cm^3^/5 min, the stage was completed. Finally, triaxial shear tests were performed under drained conditions at a shear rate of 0.042 mm/min until the axial strain reached 30%. When the shearing stage was completed, the specimen was washed into the drying plate from the rubber membrane using the water washing method, followed by a drying and sieving test. (For 0.25–0.5 mm and 0.075–0.25 mm particle size groups, the gradation curve results after the test are not shown in the gradation curve evolution section. The main reason is that some of the particles adhered to the drying plate, these sticking particles suffered a non-negligible crushing amount when removed, which was not caused by pure mechanical behavior (triaxial test), thus interfering with the test results).

## 3. Triaxial Consolidation and Drainage Test Results

### 3.1. Stress–Volume Strain–Axial Strain Characteristics

**Figure 5:**
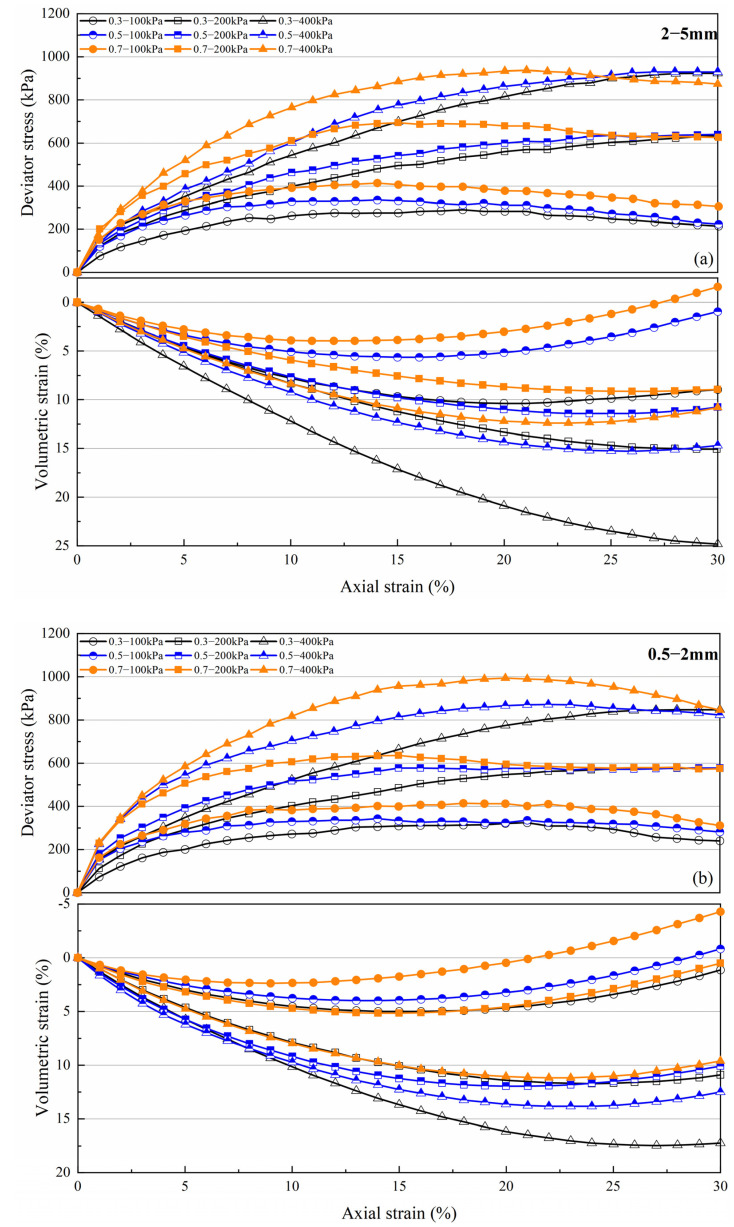

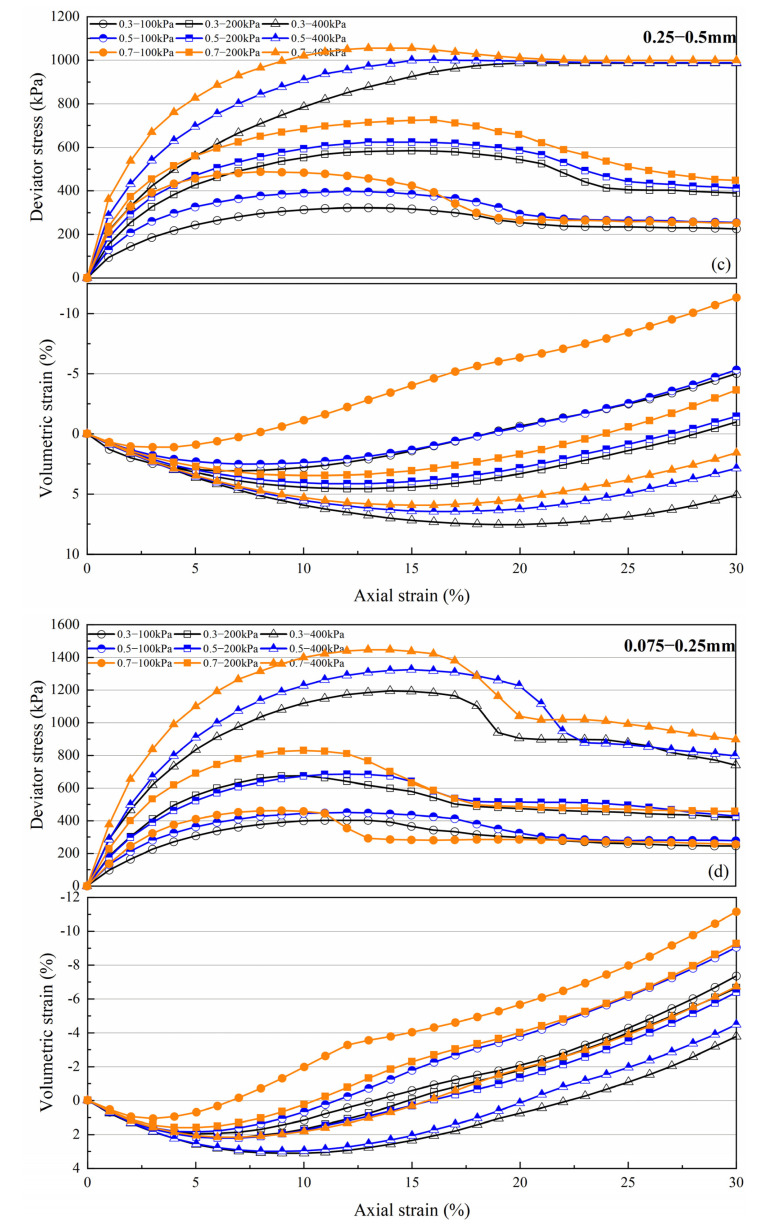
Deviator stress–axial strain–volumetric strain curves of volcanic soil with different relative density (*Dr*) and confining pressure(*σ*_3_): (**a**) 2–5 mm particle group; (**b**) 0.5–2 mm particle group; (**c**) 0.25–0.5 mm particle group; (**d**) 0.075–0.25 mm particle group.

Figure 5 shows deviator stress–axial strain–volumetric strain curves of volcanic soil with four particle size groups. Under 100 kPa confining pressure, stress–strain curves of the four particle sizes at different relative densities show a strain-softening type. For the 2–5 mm particle size group, loose samples show an initially compressive behavior, followed by an expansive behavior at low confining pressure and a purely compressive behavior at high confining pressure (400 kPa), while for the 0.075–0.25 mm particle size group, there is no pure compression behavior. For a certain particle size group, at a given confining pressure, increasing *Dr* remarkably increases the shear strength and the expansion trends, in addition, increasing confining pressure would postpone the arrival of the peak shear strength [21]. For the same particle size group, under a certain confining pressure, soil residual strengths are the same with different relative densities.

Under 30% terminating axial strain condition, four particle size groups have reached the stress critical state. For the small size group, 0.075–0.25 mm and 0.25–0.5 mm, the critical state appears to correspond to approximately 20% axial strain, while for the large size group, 0.5–2 mm and 2–5 mm, the stress critical state appears after 25% axial strain. This indicates that larger particles require greater axial shear strain to achieve stress stability.

**Figure 6:**
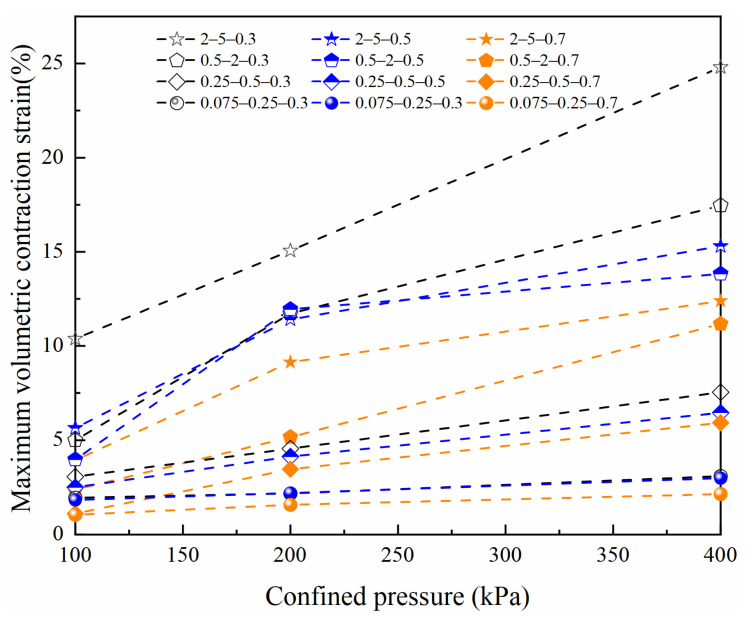
Maximum volumetric contraction strain of volcanic soil with different relative density and confining pressure in four particle size groups.

Figure 6 shows the maximum volumetric contraction strain of four particle size groups after triaxial shear testing. The maximum volumetric contraction strain of all specimens increased with increasing confining pressure, particle size, and decreasing relative density varied from 1% to 25%; under the same relative density and confining pressure conditions, as particle size increases, the void ratio also increases, and particles are more likely to move and crush during shearing, producing greater compressibility [32]. Hence, in road engineering, the selection of smaller grain size volcanic soil as road foundation fill can significantly reduce the settlement deformation of the foundation.

### 3.2. Peak Strength Index

**Figure 7:**
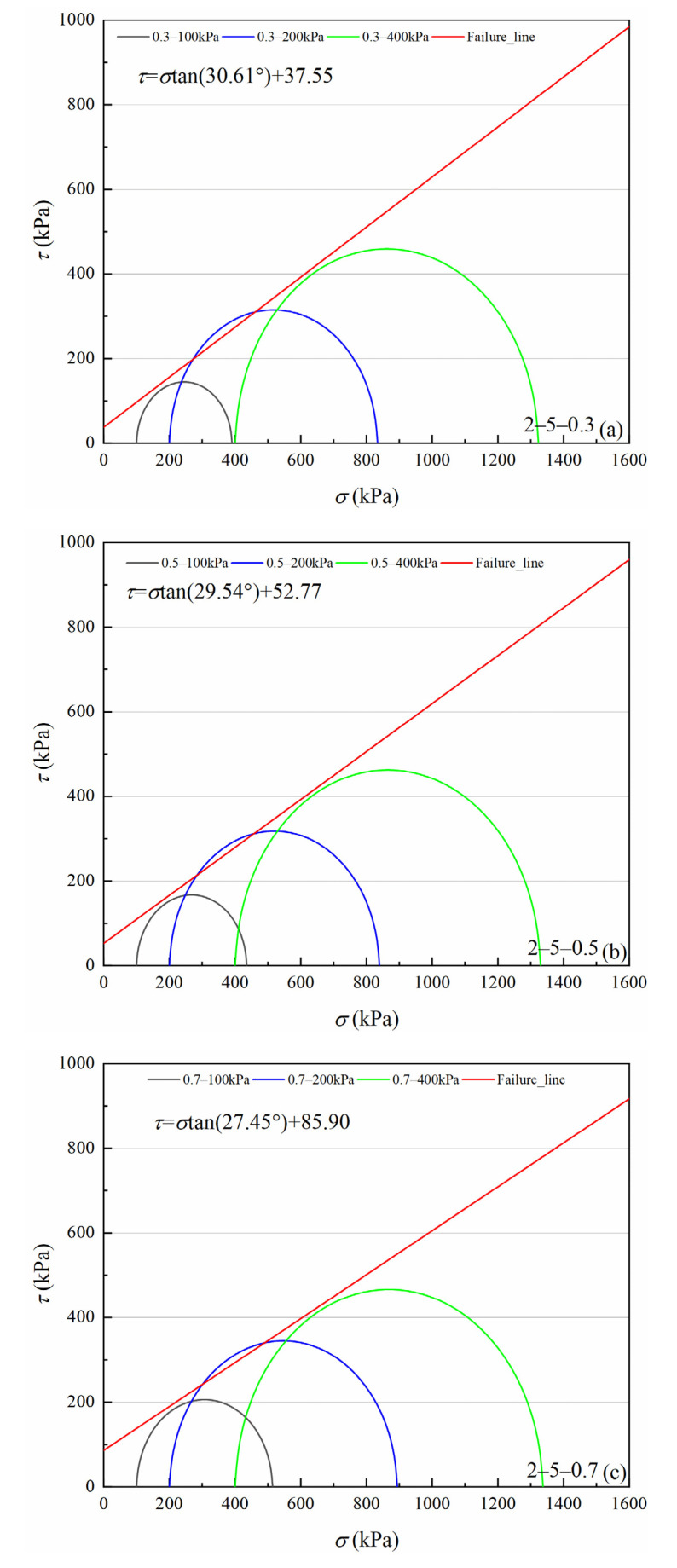
Shear strength envelopes of 2–5 mm particle groups volcanic soil with different relative density (*Dr*) under the peak state: (**a**) *Dr* = 0.3, (**b**) *Dr* = 0.5, (**c**) *Dr* = 0.7.

**Figure 8:**
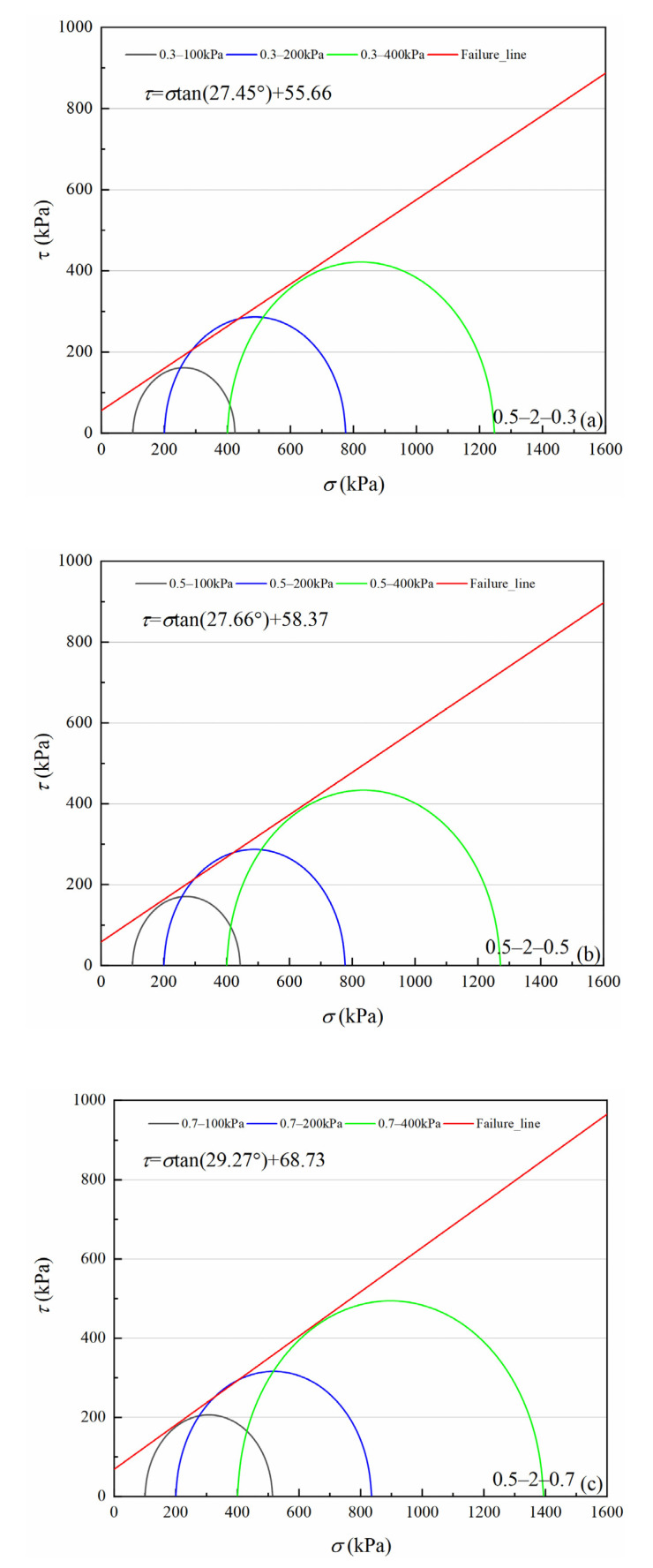
Shear strength envelopes of 0.5–2 mm particle groups volcanic soil with different relative density (*Dr*) under the peak state: (**a**) *Dr* = 0.3, (**b**) *Dr* = 0.5, (**c**) *Dr* = 0.7.

**Figure 9:**
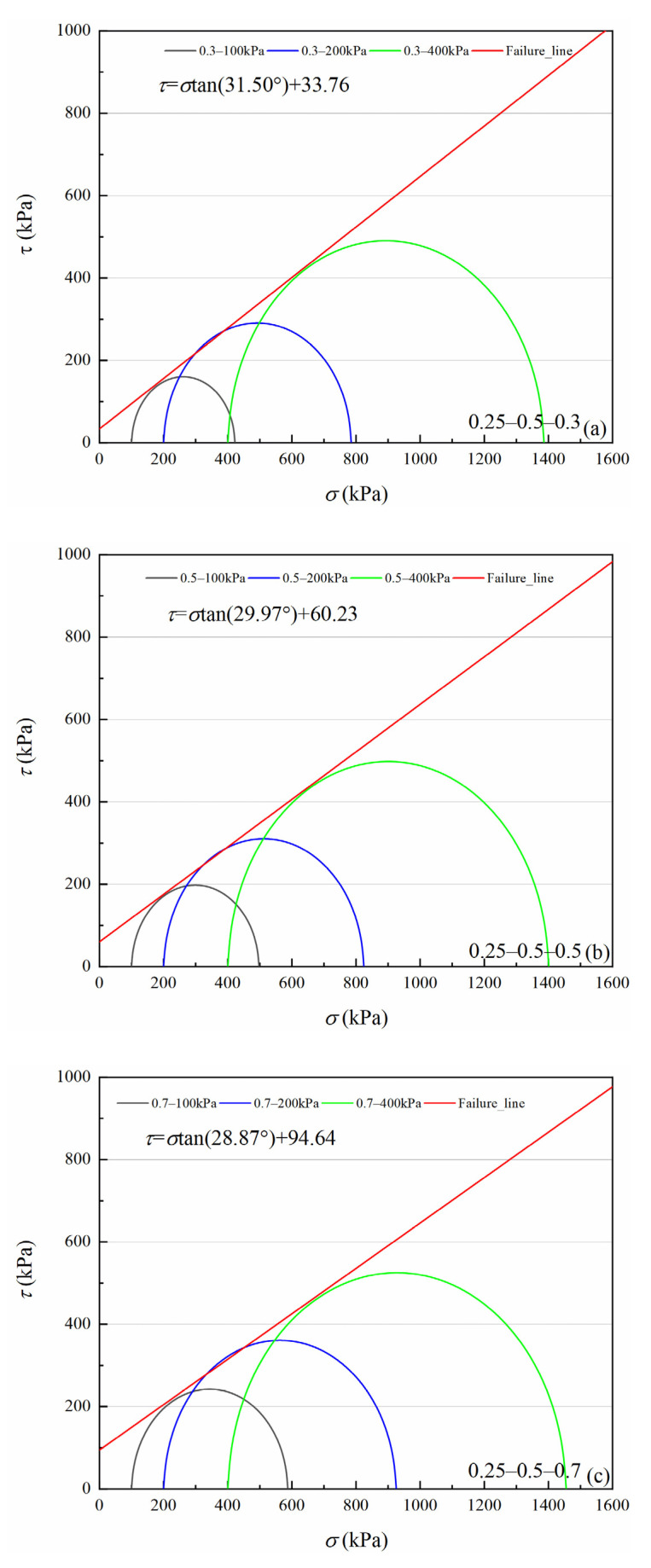
Shear strength envelopes of 0.25–0.5 mm particle groups volcanic soil with different relative density (*Dr*) under the peak state:(**a**) *Dr* = 0.3, (**b**) *Dr* = 0.5, (**c**) *Dr* = 0.7.

**Figure 10:**
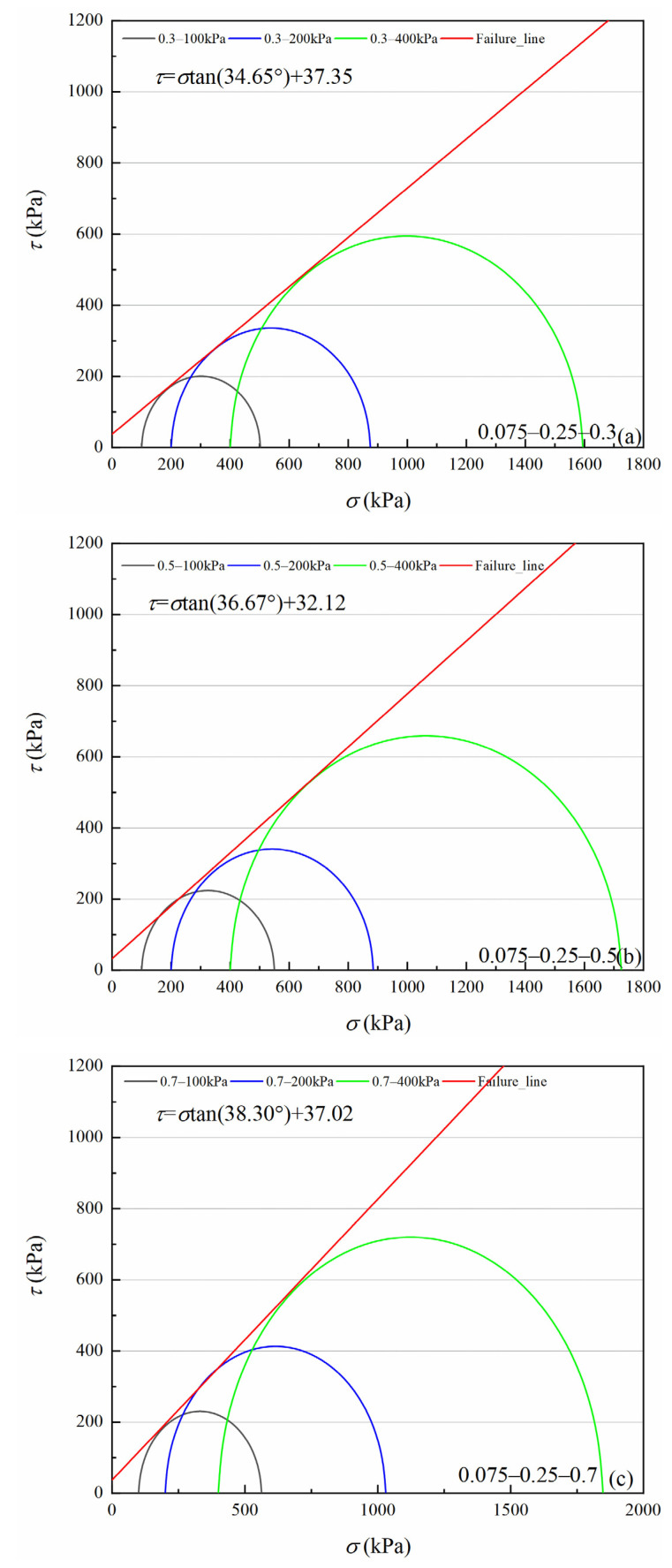
Shear strength envelopes of 0.075–0.25 mm particle groups volcanic soil with different relative density (*Dr*) under the peak state: (**a**) *Dr* = 0.3, (**b**) *Dr* = 0.5, (**c**) *Dr* = 0.7.

Figure 7, Figure 8, Figure 9 and Figure 10 show four volcanic soil particle size groups shear strength envelopes with different relative densities (*Dr*) under peak state. Although these four particle sizes belong to the coarse particle size group in the particle size category, the fitted values reveal that there is still a non-negligible cohesion, approximately 30.62–94.64 kPa, which may be attributed to the occlusion effect caused by the irregular shape of coarse particles, according to previous studies on similar materials, e.g., calcareous sands [32,33]. For the >0.25 mm particle sizes group, as for the same particle size group, cohesion increases significantly with increases in relative density. For 0.5–2 mm and 0.075–0.25 mm particle groups, the internal friction angle slowly increases with increases in relative density. With an increase in relative density, the internal friction angle tends to decrease for 2–5 mm and 0.25–0.5 mm particle size groups.

According to classical Mohr–Coulomb theory, the shear strength index is only related to the material composition and the initial density of specimens, not to the load stress level [34]. If Mohr–Coulomb fitted values are used, the internal friction angle and cohesion are artificially separated from the engineering perspective. The occlusion effect is also a part of the friction component in coarse-grained soils [29,35]. Volcanic soils can be crushed at low load stress levels. As confining pressure increases, particle crushing degree increases to suppress shear dilation behavior between particles, which leads to a decrease in the internal friction angle [36]. Therefore, it is necessary to consider the internal friction angle decreasing effect as confining pressure increases for crushable volcanic soils. For coarse-grained soils, the peak friction angle ($\varphi_{max}$) is calculated as Equation (1) [29].

(1) $$\varphi_{max}=sin^{-1}\frac{{\left(\sigma_{1}-\sigma_{3}\right)}_{max}}{{\left(\sigma_{1}+\sigma_{3}\right)}_{max}}\;$$

**Figure 11:**
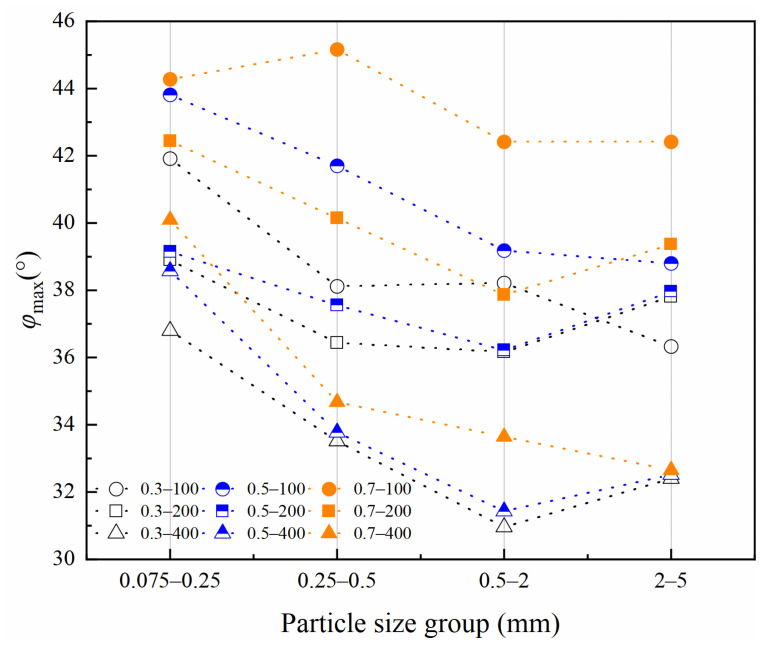
Peak internal friction angle of volcanic soil with different relative density (*Dr*) and confining pressure(*σ*_3_) in four particle size groups.

Figure 11 shows the peak internal friction angle of volcanic soil. Under the same relative density and particle size condition, peak friction angle decreases significantly with an increase in confining pressure. Under low confining pressure, the particle crushing degree is slight, and the shear dilation effect between the particles increases occlusion friction, causing a higher peak internal friction angle. With an increase of confining pressure, deviator stress increases, which causes particle crushing degree increases; particle crushing effect partially eliminates shear dilation effect, shear dilation gradually disappears, the occlusion effect is gradually eliminated, and the friction effect between particles is gradually reduced [36]. The results reveal that the peak friction angle decreases as particle size increases and relative density decreases. Therefore, in engineering construction, the greater the relative density and the higher the content of fine particles in road foundation fill, the better its bearing capacity.

### 3.3. Critical State Line

**Figure 12:**
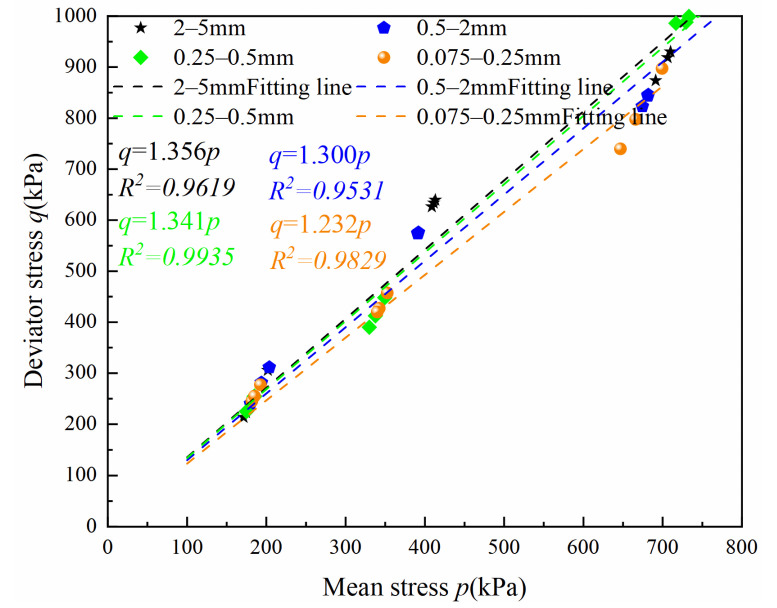
Critical state lines of volcanic soil with different particle sizes on deviator stress and mean stress plane.

According to the results in Section 3.1, four grain size volcanic soils reached critical conditions at 30% axial strain. Figure 12 shows critical state lines of volcanic soil with four different particle size groups. Critical state stress ratios of different particle size groups are significantly different. The critical state stress ratio is the largest for the 2–5 mm particle size group and the smallest for the 0.075–0.25 mm particle size group.

## 4. Gradation Curve Evolution

**Figure 13:**
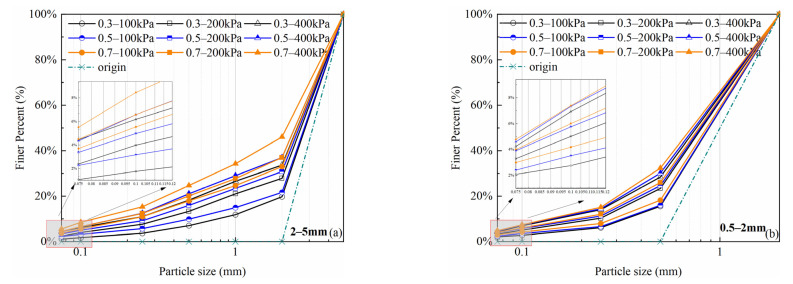
Evolutions of particle size distribution curves of volcanic soil with different relative density (*Dr*) and confining pressure(*σ*_3_): (**a**) 2–5 mm particle groups; (**b**) 0.5–2 mm particle groups.

After shearing tests, the gradation curves of both particle size groups significantly shifted upward with an increase in relative density and confining pressure (Figure 13). At 100 kPa confining pressure condition, specimen gradation curves have changed significantly, which indicates that volcanic soils have generated large crushing amounts at low confining pressures [9]. For a certain particle size group, the gradation curve shifts upward continuously with increasing relative density (*Dr*) and confining pressure(*σ*_3_). Under the same conditions, the denser specimen shows a greater crushing amount. A dense specimen will contain an increased number of grains and therefore have a larger grain coordination number. Conversely, a larger grain coordination number will enhance the interactions between the grains and the surrounding grains and increase the probability of grain crushing [37]. For different particle size groups, the crushing degree of the 2–5 mm particle size group is greater under the same confining pressure and relative density. Larger particles are more vulnerable to crushing because they contain more internal flaws and the surface shapes are more irregular. Overall, the particle crushing degree of volcanic soil is influenced by confining pressure, relative density, and particle size.

### 4.1. Crushing Degree Measurement

#### 4.1.1. Relative Breakage Rate *Br*

**Figure 14:**
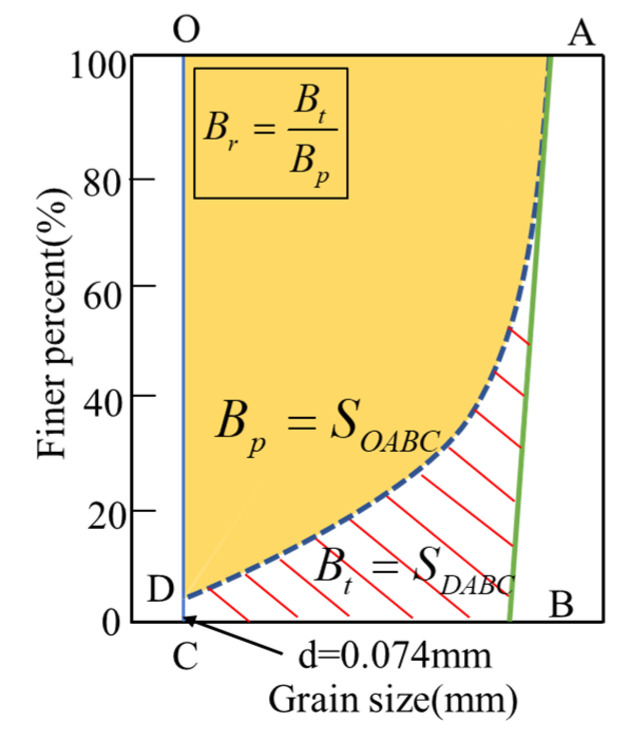
Defining of the relative breakage rate.

The relative breakage rate *Br* proposed by Hardin [11] is broadly used to evaluate the particle crushing degree in the soil mass. The definition of *Br* calculated is shown in Figure 14. Where *Bp* is defined as the area enclosed inside the initial grading curve (AB line) and the line of 0.074 mm particle size (OC line); *Bt* is defined as the area enclosed inside the current grading (AD line), initial grading (AB line), and the 0.074 mm particle size line (CD line).

**Figure 15:**
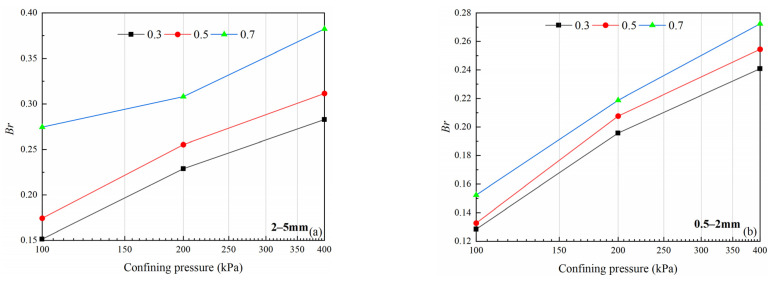
Variations in relative breakage rate (*Br*) with different relative density (*Dr*) and confining pressure(*σ*_3_): (**a**) 2–5 mm particle groups; (**b**) 0.5–2 mm particle groups.

For both particle size groups, *Br* increases with increasing confining pressure and relative density (Table 4, 2nd column), and the maximum *Br* reached 0.382. There is a positive correlation between *Br* and confining pressure (Figure 15), and the 2–5 mm particle size group has a greater relative breakage rate under the same conditions.

#### 4.1.2. Fractal Dimension *D*

Previous studies on soil particle crushing indicated that the mass distribution of crushed particles showed fractal characteristics [32,38,39,40]. The mass of particles with different sizes meets the relationship given in Equation (2).

(2) $$\frac{M(d<d_{i})}{M_{t}}={\left(\frac{d_{i}}{d_{max}}\right)}^{3-D\;}$$

where *d* is particle diameter, *d_i_* is ith sieving diameter (*i* = 1, 2, …, n); *d_max_* is the diameter of the largest particle, *M* (*d < d_i_*) is the cumulative mass of soil particles with particle size less than *d_i_*; *M_t_* is the total mass of soil particles. *D* = 3 − *k*, where *D* is fractal dimension, *k* is the slope of the relationship curve between *M*(*d* < *d_i_*)/*M_t_* and *d_i_*/*d_max_* in the logarithmic coordinate system.

**Figure 16:**
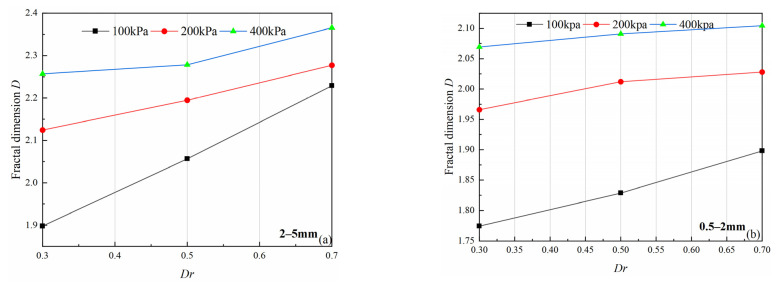
Variations in fractal dimension (*D*) with different relative density (*Dr*) and confining pressure(*σ*_3_): (**a**) 2–5 mm particle groups; (**b**) 0.5–2 mm particle groups.

Before tests, these two particle size groups (0.5–2 mm, 2–5 mm) do not show fractal characteristics; after shear tests, all specimens exhibit obvious fractal characteristics, (Table 4, 3th column). Fractal dimension *D* increases with an increase of confining pressure *σ*_3_ and relative density *Dr*, and the fractal dimension shows a positive correlation with relative density (Figure 16). Under the same conditions, the 2–5 mm particle size group has a larger fractal dimension. This is because the initial particle size of that is larger, and particle crushing produces a wider particle size distribution, which makes fractal characteristics more obvious [32].

#### 4.1.3. Associations between *Br* and *D*

**Figure 17:**
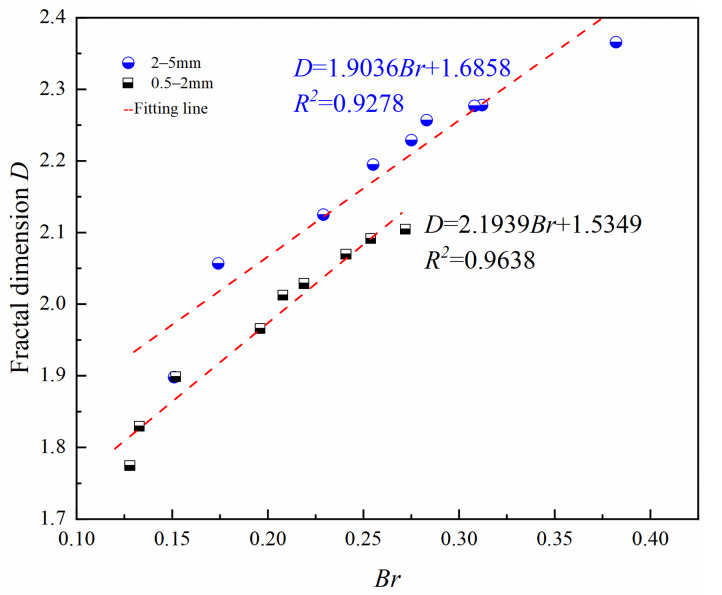
Variations in Fractal dimension (*D*) with relative breakage rate (*Br*).

Figure 17 shows the variation of fractal dimension *D* with relative breakage rate *Br*; there is a good linear relationship between them, and the relationship between *D* and *Br* is not affected by relative density and confining pressure only by particle size. The 2–5 mm particle size group has a larger fractal dimension compared with the 0.5–2 mm particle group under the same *Br*.

### 4.2. Particle Crushing Pattern

Soil particle crushing patterns are generally classified into three patterns: abrasion, breakoff, and splitting [41]. Dong et al. [42] proposed and experimentally verified a probability density function that can describe a single particle size group crushing pattern based on a large number of point load experiments. The Hill probability density function f is expressed as follows:

(3) $$f=-\frac{a^{b}b{\left(\frac{x_{i}}{1-x_{i}}\right)}^{b}}{x_{i}\left(x_{i}-1\right){\left[{\left(\frac{x_{i}}{1-x_{i}}\right)}^{b}+a^{b}\right]}^{2}}\;$$

where $x_{i}=d_{i}/d_{max}$, *d_i_* is crushed sub-particle size, and *d_max_* is the maximum particle size of the original particle group. The fitting parameters are *a* and *b*. Parameter *a* controls the particle size ratio that is most likely to appear after particle crushing, and parameter *b* controls the curve shape. According to the parameters of *a*, *b*, three crushing patterns can be determined. In this paper, values *a* range from 0.68 to 3.173, and values *b* range from 0.64 to 1 (Table 4, 5th, 6th column).

**Figure 18:**
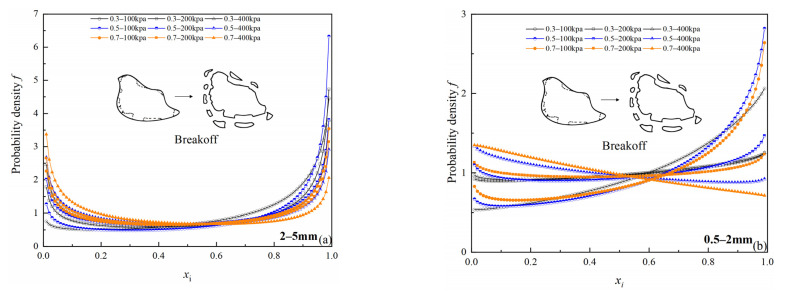
Variation of Hill probability density function f with different relative density (*Dr*) and confining pressure (*σ*_3_): (**a**) 2–5 mm particle groups; (**b**) 0.5–2 mm particle groups.

**Figure 19:**
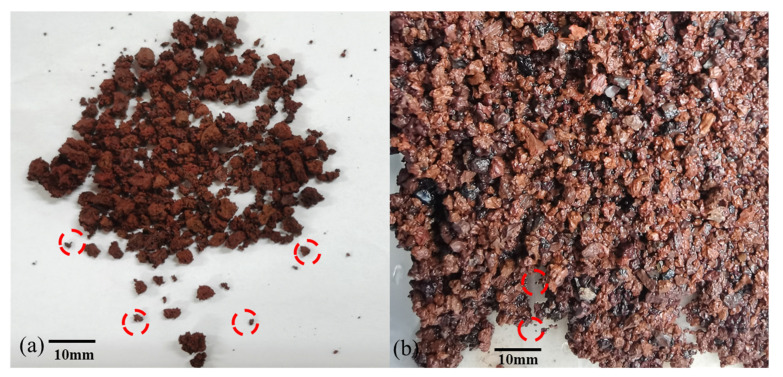
Photos of volcanic soil after shear test: (**a**) 2–5 mm-0.7–400 kPa test sample; (**b**) 0.5–2 mm–0.7–400 kPa test sample.

Figure 18 shows the variation of Hill probability density function *f* in two particle size groups: for any one of the specimens, as *x_i_* increases, the probability density function *f* presents that extreme values appear at both ends (*x_i_* near 0 or 1) of the curve, and one end is higher than the other; according to Dong’s result [42], the curve types of these two particle size groups can be defined as breakoff type, which is in good agreement with the test photographs (Figure 19). For the same particle size groups, when *x_i_* is near zero, the probability density function *f* shifts upward as confining pressure increases under the same relative density condition. However, there is no obvious rule for the probability density function *f* at different relative densities under the same confining pressure, which indicates that the Hill probability density function may not be sensitive to the change in the relative density.

## 5. Plastic Work Analysis

Since grain crushing is a process in which grains consume external work and result in energy loss, the evolution laws of volcanic soil grain crushing during the test can be studied in terms of plastic work (*Wp*) [37,43]. *Wp* is defined as the irrecoverable energy extracted by the sample during the test, and it is equal to the sum of plastic work done by shear stress (*W*_1_) and the plastic work done by mean effective stress (*W*_2_). Given that crushable coarse-grained soils undergo only slight elastic deformation during the test, dεd and dεv could be directly adopted to determine the *Wp* [44].

(4) $$Wp=W_{1}+W_{2}=\int qd\varepsilon_{d}^{p}+\int pd\varepsilon_{v}^{p}\approx \int qd\varepsilon_{d}+\int pd\varepsilon_{v}\;$$

**Figure 20:**
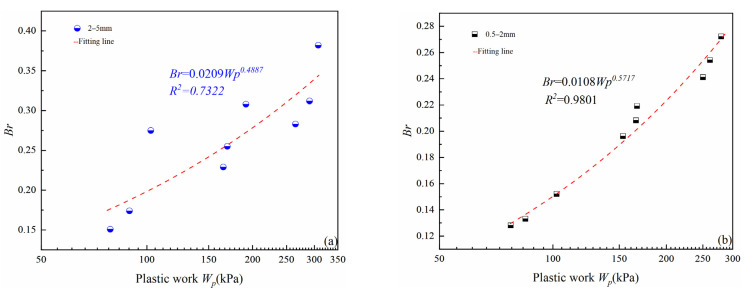
Variations in relative breakage rate with plastic work: (**a**) 2–5 mm particle groups; (**b**) 0.5–2 mm particle groups.

The results of plastic work for these two particle sizes group are shown in Table 4 (8^th^ column); for the same particle size group, plastic work increases with the increase of confining pressure and relative density. For different particle size groups, the 2–5 mm particle size group needs to consume more plastic work, whereas the difference is not large. Under the same confining pressure and relative density conditions, for example, plastic work consumed by 2–5 and 0.3–100 kPa specimens and 0.5–2 to 0.3–100 kPa specimens are basically the same, but the difference between their relative breakage rate *Br* was larger, which indicates that the 0.5–2 mm particle group needs more plastic work than the 2–5 m particle group to reach the same relative breakage rate *Br*. The main reason is that the shape of the 2–5 mm particle group is more irregular, with larger internal flaws compared to the 0.5–2 mm particle group (Figure 4), which leads to a greater susceptibility to crushing for the same external load work conditions, causing a greater relative breakage rate. The relationship between the plastic work (*Wp*) and the relative breakage rate (*Br*) for these two particle sizes groups follows the power function (Figure 20a,b), which can be fitted using Equation (5), with *A*, *B* as the fitting parameters. For the same particle size groups, the relationship between plastic work and the relative breakage rate is not significantly influenced by the relative density and the confining pressure. Scholars’ studies on calcareous sands also confirm this conclusion [37], however, in their research,$\;Br=1.17\times 10^{-5}Wp^{1.42}$. By comparing the equations in this study with theirs, it can be found that the amount of crushing is greater under the same plastic work in this study, which is mainly because the main minerals strength of calcareous sand are stronger compared to volcanic soil particles, therefore, calcareous sand particles are less likely to crush under the external force, which also shows that the mineral composition has a great influence on particle breakage.

(5) $$Br=AWp^{B}$$

## 6. Conclusions

A series of consolidation drainage shear tests were carried out to investigate the effects of particle size, confining pressure, and relative density on the mechanical properties and crushing characteristics of volcanic soils. The characteristics of soil stress–strain curve, shear strength index, critical state behavior, and quantification of particle fragmentation characteristics, using fractal dimension and relative breakage rate were systematically investigated. Finally, the quantitative relationship between external work and fragmentation amount was established through the energy perspective. The main findings are as follows:

Stress–strain curves characteristic of volcanic soil were affected by particle size; the large particle size groups (>0.5 mm) required larger axial strain to reach a stress steady state. The critical stress ratio was significantly influenced by particle size, with the largest critical stress ratio for the 2–5 mm particle size group and the smallest critical stress ratio for the 0.075–0.25 mm particle size group.

For crushable volcanic soils, there was a clear physical significance in using the peak internal friction angle index when considering the confining pressure effect; peak friction angle decreased significantly with the increase of confining pressure, and peak friction angle decreased as particle size increased and relative density decreased.

The particle crushing amount increased with increasing particle size, relative density, and confining pressure. For the same particle size, there was a good linear relationship between *Br* and *D*. Crushing patterns of large particle size groups could be classified as breakoff type. From an energy perspective, the power function relationship between *Wp* and *Br* of large particle size group was established, the relationship was only affected by particle size, and it was not significantly affected by the relative density and effective confining pressure. The results of comparison with similar materials [37] also confirm that the mineral composition has a large influence on the particle crushing amount.

In terms of engineering, based on the results of this study, it can be concluded that volcanic soil with fine particles used as roadbed filler can significantly reduce the deformation of the roadbed and improve the bearing capacity of the foundation, in addition to increasing the compaction degree of volcanic soil.

## Figures and Tables

**Table 1:** Proportions of primary minerals and major oxides for samples.

Test item	Component	Percentage (%)	Test Item	Component	Percentage (%)
XRD mineral phase	Quartz	20.5	XRF major element	SiO_2_	50.18
				Al_2_O_3_	16.95
	Potassium feldspar	20.6		Fe_2_O_3_	10.48
				FeO	0.77
	Plagioclase	48.9		MgO	2.27
				CaO	4.39
	Hematite	8.5		Na_2_O	4.13
				K_2_O	3.87
	Clay minerals	1.5		MnO	0.18
				P_2_O_5_	1.23
				TiO_2_	1.75
				Loss on ignition	3.53

**Table 2:** Physical parameters of volcanic soil.

Soil Group (mm)	Soil Name	*G*s	*ρ*_dmin_ (g/cm^3^)	*ρ*_dmax_ (g/cm^3^)	e_max_	e_min_
2–5	Fine gravel	2.615	0.597	1.277	3.380	1.048
0.5–2	Coarse sand		0.729	1.262	2.587	1.072
0.25–0.5	Medium sand		0.875	1.361	1.989	0.921
0.075–0.25	Fine sand		0.930	1.391	1.811	0.879

**Table 3:** Triaxial shear test scheme for four particle size group.

Type of Test	Soil Group (mm)	*Dr* (Relative Density)	Confining Pressure (kPa)	Total Number of Samples
CD Test	2–5	0.3 (loose), 0.5 (medium), 0.7 (dense)	100, 200, 400	36
	0.5–2			
	0.25–0.5			
	0.075–0.25			

**Table 4:** Table of correlation coefficients and plastic work for large particle size group.

Sieving Test Sample	*Br*	*D*	*R*^2^ (Fractal)	*a*	*b*	*R*^2^ (HILL)	*Wp* (kPa)
2-5-0.3-100	0.151	1.898	0.958	3.173	0.812	0.988	78.599
2-5-0.3-200	0.229	2.125	0.969	2.275	0.658	0.982	165.014
2-5-0.3-400	0.283	2.257	0.971	1.456	0.642	0.980	264.891
2-5-0.5-100	0.174	2.057	0.929	3.902	0.656	0.990	89.152
2-5-0.5-200	0.255	2.195	0.970	1.674	0.677	0.978	169.370
2-5-0.5-400	0.312	2.278	0.977	1.071	0.677	0.970	290.556
2-5-0.7-100	0.275	2.229	0.971	1.442	0.671	0.973	102.541
2-5-0.7-200	0.308	2.277	0.978	1.153	0.664	0.978	191.436
2-5-0.7-400	0.382	2.366	0.988	0.681	0.697	0.983	307.688
0.5-2-0.3-100	0.128	1.774	0.988	2.015	0.984	0.991	77.369
0.5-2-0.3-200	0.196	1.965	0.996	1.174	0.979	0.991	153.606
0.5-2-0.3-400	0.241	2.069	0.996	1.146	0.972	0.989	250.489
0.5-2-0.5-100	0.133	1.829	0.981	2.274	0.905	0.984	84.601
0.5-2-0.5-200	0.208	2.012	0.996	1.172	0.931	0.990	166.295
0.5-2-0.5-400	0.254	2.091	0.995	0.822	0.970	0.989	261.325
0.5-2-0.7-100	0.152	1.898	0.985	1.974	0.883	0.988	102.395
0.5-2-0.7-200	0.219	2.028	0.997	1.048	0.952	0.992	167.419
0.5-2-0.7-400	0.272	2.104	0.995	0.724	1.005	0.992	279.888

## Data Availability

Data is contained within the article.

## Abbreviations

*Gs*	Specific gravity
*ρ_dmin_*	Minimum dry density (Unit: g/cm^3^)
*ρ_dmax_*	Maximum dry density (Unit: g/cm^3^)
*e_min_*	Minimum void ratio
*e_max_*	Maximum void ratio
*d*	Particle size (mm)
*d*_30_	The mass of soil particles smaller than this particle size is 30% of the total mass of soil particles (mm)
*d*_60_	The mass of soil particles smaller than this particle size is 60% of the total mass of soil particles (mm)
*Dr*	Relative density
*σ*_3_	Confining pressure (Unit: kPa)
*σ*	Normal stress (Unit: kPa)
*τ*	Shear stress (Unit: kPa)
*c*	Soil cohesion (Unit: kPa)
*φ*	Soil internal friction angle (°)
*D*	Fractal dimension
*Br*	Relative breakage rate
*R* ^2^	Coefficient of correlation
*f*	Probability density function
*W_p_*	Plastic work (Unit: kPa)
*W*_1_	Plastic work done by shear stress (Unit: kPa)
*W*_2_	Plastic work done by mean effective stress (Unit: kPa)
*q*	Deviator stress (Unit: kPa)
*p*	Mean effective stress (Unit: kPa)
$d\varepsilon_{d}^{p}$	Plastic shear strain increment
$d\varepsilon_{v}^{p}$	Plastic volumetric strain increment
$d\varepsilon_{d}$	Shear strain increment
$d\varepsilon_{v}$	Volumetric strain increment
*a*	Fitting parameter
*b*	Fitting parameter
*A*	Fitting parameter
*B*	Fitting parameter
